# The Association between Malnutrition and Pressure Ulcers in Elderly in Long-Term Care Facility

**DOI:** 10.3889/oamjms.2016.094

**Published:** 2016-08-22

**Authors:** Lenche Neloska, Katerina Damevska, Andjelka Nikolchev, Lidija Pavleska, Biljana Petreska-Zovic, Milenko Kostov

**Affiliations:** 1 *Gerontology Institute “13 November Skopje”, Skopje, Republic of Macedonia*; 2 *University Clinic of Dermatology, Medical Faculty, Ss Cyril and Methodius University of Skopje, Skopje, Republic of Macedonia*; 3 *University Clinic of Neurosurgery, Medical Faculty, Ss Cyril and Methodius University of Skopje, Skopje, Republic of Macedonia*

**Keywords:** malnutrition, pressure ulcers, geriatric patients, palliative patients, long-term care facility

## Abstract

**BACKGROUND:**

Malnutrition is common in elderly and is a risk factor for pressure ulcers.

**AIM:**

The aim of the present study was to determine the prevalence of malnutrition in geriatric and palliative patients hospitalised in long-term care facility, and to examine the influence of nutritional status on the prevalence of pressure ulcers (PU).

**MATERIAL AND METHODS:**

Descriptive, observational and cross-sectional study including 2099 patients admitted to the Hospital during a 24 month period (January 2013 to December 2014). We recorded: demographic data, body mass index (BMI), Braden score, laboratory parameters of interest (albumin, total protein, RBC count, haemoglobin and iron levels) and presence or absence of malnutrition and pressure ulcers.

**RESULTS:**

The pressure ulcer prevalence was 12.9% (256 out of 2099). Based on the BMI classification, 61.7% of patients had a good nutritional status, 27.4% were undernourished, and 2.1% were considered malnourished. Nutritional status was statistically significantly different between patients with and without PU (p < 0.0001). This study also showed that hypoproteinemia, hypoalbuminemia, low RBC was positively associated with PU prevalence.

**CONCLUSION:**

The results highlight the impact of nutritional status on the prevalence of pressure ulcers in hospitalised geriatric and palliative population. It is of paramount importance to correctly evaluate the presence of malnutrition in patients at risk of pressure ulcers.

## Introduction

A pressure ulcer (PU) is localised injury to the skin and/or underlying tissue usually over a bony prominence, as a result of pressure or pressure in combination with shear. It leads to ischemia, progressive destruction and necrosis of the underlying soft tissues [1]. Pressure ulcers are debilitating chronic wounds, which occur in people with advanced age, physical or cognitive impairments, and multiple comorbidities. It is a common problem among older adults in all health care settings [2].

The prevalence of PUs in long-term settings ranges from 11 to 29% [3-6]. Development of PU in hospitalised elderly patients is complex and multifactorial. Predisposing factors are classified as intrinsic (e.g. activity or mobility limitation, altered consciousness, abnormalities in nutritional status, comorbidities, ageing skin) or extrinsic (e.g., pressure, friction, shear, incontinence).

Malnutrition is a very common problem that affects approximately 30-50% of hospitalised patients. It is estimated that at least one-third of patients has some degree of malnutrition upon admission to the hospital (7). Additionally, among patients who are not malnourished upon admission, about one third may become malnourished while in the hospital (8). Among the elderly residents of long-term care institutions, the prevalence of in-hospital malnutrition has been estimated to be between 12.5 and 78.9% in different studies [9-11]. Overall, 50% of the residents require an individualised nutritional care plan [9].

Hospital malnutrition is associated with an increase in morbidity, mortality, a higher readmission rate, functional disabilities and physical complications, and, therefore, higher healthcare costs. Malnutrition is closely related to frailty, a clinical syndrome characterised by increased vulnerability to adverse health outcomes including acute illness, the decline in physiological reserve, and increased risk for disability, falls, hospitalisation, need for long-term care, and death [12].

The principal objective of this study was to assess the prevalence of malnutrition in elderly patients hospitalised in the long-term care facility. The secondary objective was to examine the influence of malnutrition on the prevalence of pressure ulcers.

## Material and Methods

This observational study was carried out at the Geriatric and Palliative Care Hospital “13 November” in Skopje, Macedonia, the largest specialised geriatric and palliative hospital in the country that attends to patients through the Public Health System. The study design was approved by the Hospital Ethics Committee. Informed consent was waived because of the study’s observational nature.

Consecutive patients admitted to the hospital were enrolled from January 2013 to December 2014. We collected data using a case report form and recorded information about demographics, main diagnosis, BMI, laboratory findings, the presence of PU and PU characteristics.

Body mass index (BMI) was calculated using the formula: weight in kilogrammes/height in meters2. Using BMI classification, patients were classified as severely underweight, underweight, normal weight, overweight, severely obese or morbidly obese.

The Braden scale was used to assess the risk of developing PUs. The total score can range from 6 to 23 with a lower score indicating a higher risk [13].

The risk for PU development was assigned according to the stratification determined by the scale, into four groups; according to the Braden score [13]. The risk of PUs increases in patients with a score ≤ 12 points. The grading system of the EPUAP (European Pressure Ulcer Advisory Panel) was used [1].

Descriptive statistics was used to describe the study population, with continuous outcomes summarised as a mean and range, and categorical outcomes presented as a percentage. Chi-square test was used to analyse categorical variables. Independent t-test was used to analyse continuous variables. P-values < 0.05 were considered statistically significant.

## Results

During the study period, two thousand and ninety-nine patients were consecutively admitted. Baseline characteristics of the study population are shown in Table 1. One thousand eight hundred and forty-three patients without PU (724 male and 1119 female, mean age 76.32 years, SD 11.192, range 22-103 years), and 256 patients with PU (86 male and 170 female, mean age 76.38, SD 11.296, range 37-97 years) participated in the study. No statistically significant difference was noted regarding age between the groups (p = 0.80).

**Table 1:** Baseline characteristics of patient population, according to the presence/absence of PU

	GENDER			AGE	

		N (%)	X̄	SD	Min.-Max.
PU absent	Male	724 (39.28)	74.63	11.697	25-103
	Female	1119 (60.71)	77.41	10.717	22-101
	Total	1843 (87.8)	76.32	11.192	22-103
PU present	Male	86 (33.59)	74.50	12.234	38-97
	Female	170 (66.4)	77.34	10.702	37-95
	Total	256 (12.19)	76.38	11.296	37-97
Total		2099 (100)	76.32	11.202	22-103

Braden score ranged from 6 to 23 (mean 13.64, SD 3.247) and 1948 (92.8%) had a risk for pressure ulcers (Table 2).

**Table 2:** Patient’s level of risk for development of PUs according to the Braden Scale (BS)

Level of risk	N	%
High (BS ≤ 12)	913	43.5
Moderate (BS 13-14)	478	22.8
Low risk (BS 15-19)	557	26.5
No risk (BS ≥ 20)	151	7.2
Total	2099	100

Table 3 shows the distribution of patients according to the nutritional status. Based on the BMI classification, 61.7% of patients had a good nutritional status, 27.4% were undernourished, and 2.1% were considered malnourished. Nutritional status was statistically significantly different between patients with and without PU (χ^2^ = 25.350; p < 0.0001).

**Table 3:** Differences in nutritional status between patients with (n = 256) and without a pressure ulcer (n = n=1843)

BMI classification	BMI	PU absent N (%)	PU present N (%)	Total N (%)
Severely underweight	15-16	29 (1.57)	16 (6.25)	45 (2.1)
Underweight	16-18.5	501 (27.18)	74 (28.9)	575 (27.4)
Normal weight	18.5–24.9	1151 (62.45)	144 (56.25)	1295 (61.7)
Overweight	25.0–29.9	102 (5.53)	12 (4.68)	114 (5.4)
Severely obese	30.0–34.9	59 (3.2)	10 (3.9)	69 (3.3)
Morbidly obese	>35	1 (0.05)	0	1 (0.05)
Total		1843 (87.81)	256 (12.19)	2099

Table 4 shows nutrition-related laboratory values, according to the presence of PU. We detected a significant difference in presence of hypoalbuminemia (p < 0.0001), hypoproteinemia (p = 0.019), low RBC (p = 0.004) and low hemoglobin levels (p < 0.0001) in patients with PU, compared to patients without PU. Iron and triglyceride levels were not related to the presence of PU.

**Table 4:** Nutrition-related laboratory values

	PU absent	PU present	χ^2^	p
	N (%)	N (%)		
↓ RBC count	755 (40.96)	131 (51.17)	11.124	0.004
↓ Hemoglobin level	1050 (57.36)	186 (72.94)	27.156	<0.0001
↓ Iron (serum)	1 (0.07)	1 (0.5)	2.48	0.289
↓ Total protein	1194 (71.71)	197 (78.8)	5.495	0.019
↓ Albumin	704 (48.55)	175 (70.28)	40.184	<0.0001
↑Triglyceride level	220 (14.58)	34 (14.78)	0.006	0.938

## Discussion

In this retrospective study involving 2099 geriatric and palliative patients in a long-term setting, the following variables were significantly more frequently documented in patients with PU compared to those without PU: malnutrition measured as BMI, hypoproteinemia, hypoalbuminemia and anaemia (p < 0.05 for all).

**Figure 1:**
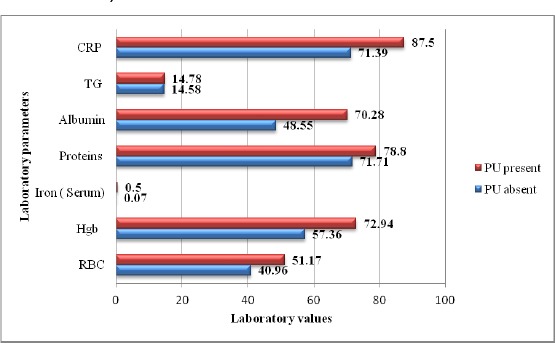
Percentage of patients with altered nutrition-related lab values

Malnutrition is defined as a state of nutrition in which a deficiency or excess of energy, protein and other nutrients causes measurable effects both on tissue/body structure and function [14].

The causes of malnutrition are multiple and complex [15]. In elderly patients, malnourishment may result from a combination of factors, including cardiac failure, difficulty chewing, dysphagia inflammatory illness, respiratory problems, reduced sense of smell and taste, and medications [16]. Furthermore, chronic diseases and cancer cause inflammation and increased cytokine production, which suppresses appetite. Acute and chronic infections, wounds, and hypermetabolism cause increased the need for energy and protein [17, 18]. Monotony of diet in institutional care, the timing of meals, decreased taste thresholds and a loss of olfactory distinction also impact nutritional wellness [19].

Malnutrition has numerous effects, including impaired collagen synthesis, and immune function. Wound healing refers to the complex and dynamic process of restoring cellular structures and tissue layers. In each phase of wound healing, poor nutritional status can delay the healing process or cause inadequate healing when nutritional deficiencies are not corrected [20, 21].

A number of studies have demonstrated that the relationship between malnutrition and pressure ulcers is bidirectional [22-24]. A direct correlation between malnutrition severity and the pressure ulcers has been reported [24].

Patients with chronic PUs - wounds that remain unhealed for more than six weeks - experience a continuous cycle in which they lose protein through excess exudate, resulting in delayed wound healing.

Our results revealed marked hypoproteinemia and hypoalbuminemia in patients with PUs. Protein-energy malnutrition reduces fibroblastic cellular activity and delays angiogenesis in the proliferative stage and reduced collagen synthesis and maturation in the remodelling stage, leading to increased wound dehiscence. Furthermore, proteins are lost in wound exudates, which can contain as much as 100 g of protein per day [25]. A deficiency in serum albumin causes impaired wound perfusion, reducing the osmotic pressure in the intravascular space. This causes interstitial oedema, reducing tissue oxygenation and tissue tolerance to the forces of pressure. Oedema also may be a factor in unidentified malnutrition by masking muscle and fat loss [26].

Older people are at risk of malnutrition if they have a BMI below 24, and malnutrition is indicated by a BMI of less than 20 [27]. The World Health Organization categorises underweight as BMI < 18.5. However, BMI may be unreliable in the presence of confounding factors such as oedema or ascites, and may not identify significant unintentional weight loss if used as a single assessment. Reliable measurement of height can be difficult in the elderly because of vertebral compression, loss of muscle tone and postural changes [28].

Malnutrition in a hospital setting may be prevented by using strategies that include assisting patients into a position conducive to swallowing safely and comfortably; implementing food charts to ensure accurate documentation of intake; and using fluid charts to record input and output to calculate fluid balance [21].

Maintaining nutritional status and preventing malnutrition aims to ensure that energy, protein and other micronutrients are available to prevent pressure ulcer development. No biochemical marker on its own offers a satisfactory screening test for malnutrition. Serum proteins synthesised by the liver have been used as markers of nutrition—albumin, transferrin, retinol-binding protein and thyroxine-binding albumin. Serum albumin has been most widely adopted; however, the long half-life of albumin means that serum albumin does not respond to short-term changes in protein and energy intake.

Nutrition and hydration play an important role in preserving skin and tissue viability and in supporting tissue repair for PU healing [29].

In the older population, undernutrition rather than overnutrition is the main cause for concern [30]. Malnourished older people are at increased risk of falls, lengthy hospital stays and rehabilitation, institutionalisation, postoperative complications, infections, pressure ulcers, poor wound healing, impaired muscle and respiratory function and death [31]. Unfortunately, malnutrition continues to be under-recognized in many hospitals [32].

Pressure ulcers are debilitating chronic wounds. Prevention of PUs in hospitalised elderly patients is an important health priority, one that requires clear identification of risk factors. Effective management of malnutrition requires collaboration among multiple clinical disciplines and all members of the clinical team [33].

In conclusion, malnutrition is a problem of high prevalence and impact in geriatric and palliative patients. Given the high prevalence of malnutrition among patients with pressure ulcers, performing a routine nutritional screening should result in early identification of residents with the risk of development of pressure ulcer.
